# The Awareness and Practice of Self-Medication Among the General Public in Jeddah and Makkah

**DOI:** 10.7759/cureus.39706

**Published:** 2023-05-30

**Authors:** Syed F Zaidi, Alqassem Y Hakami, Muhammad A Khan, Adil A Khalid, Ahmed K Haneef, Safwan S Natto, Mohammed A Mastour, Rayan F Alghamdi

**Affiliations:** 1 College of Medicine, King Saud Bin Abdulaziz University for Health Sciences, Jeddah, SAU; 2 Faculty of Eastern Medicine, Hamdard University Islamabad Campus, Islamabad, PAK; 3 Medical Research, King Abdullah International Medical Research Center, Jeddah, SAU; 4 Medical Education, King Saud Bin Abdulaziz University for Health Sciences, Jeddah, SAU

**Keywords:** self-medication, knowledge among pharmacists, general public health, medication side-effects, over-the-counter drugs

## Abstract

Background

Self-medication (SM) can be defined as the improper practice of obtaining and consuming a pharmaceutical drug without the consultation or prescription of a licensed physician. This includes evaluating the intensity of signs and symptoms which could lead to treating oneself with a medicine or seeking urgent medical care. Although SM can be deemed as safe for one’s health, drug accessibility accounts for an irrational choice of medicines and thus exposes oneself to the adverse effects of these medicines. Several regional studies have provided sufficient evidence about how SM has commonly been practiced and held in some settings, such as pharmacies.

Aim

In this study, we aimed to assess the practice and awareness of SM in the general public. Thus, we utilized a questionnaire-based survey to analyze SM awareness and practice among the general population in Jeddah and Makkah. In addition, we examined the impact of demographic variables, such as educational level, economic status, age, etc. on SM practice.

Methods

A cross-sectional survey was distributed via social media platforms in June 2020. The study included Jeddah and Makkah's general public, all different nationalities, and both genders, and it excluded participants below the age 18-year-old and with mental and cognitive instability. After extrapolating the sample size at a 95% confidence level with an estimated 50% response distribution, a margin of error ±5%, and accounting for a 5% non-response rate, the estimated sample size was determined to be 404.

Results

A total of 642 participants completed the online-based survey, but only 472 responses fit the study criteria. Most of the participants (64.6%) did not consult with a physician, i.e., practiced SM, whereas (34.5%) have visited a doctor. Furthermore, people who did not visit a doctor had the commonest belief (26.1%) that they did not need a doctor to investigate their symptoms. The awareness of SM among the general public in Makkah and Jeddah was assessed by asking whether they deem this practice harmful, harmless, or beneficial. 65.9% of the participants deemed the practice of SM as harmful, and 17.6% regarded the act as harmless.

Conclusion

This study revealed that 64.6% of the general public of Jeddah and Makkah practice self-medication, even though 65.9% deemed this act harmful. The contradiction between the public's opinion and the actual behavior towards self-medication implies the need for more awareness of self-medication and the importance of exploring the incentives of such behavior.

## Introduction

Self-medication (SM) can be defined as the improper practice of obtaining and consuming a pharmaceutical drug without the consultation or prescription of a licensed physician. This includes evaluating the intensity of signs and symptoms which could lead to treating oneself with medicine or seeking urgent medical care [1]. Drug accessibility has been emphasized by the World Health Organization (WHO) to ensure patients’ medical needs. This creates a community that unintentionally as a whole practices SM [2]. On a smaller scale, family members have access to their home pharmacy; unfortunately, it has been found that even children can find access to their parents' medicines in their households [3]. Although SM can be deemed safe for one’s health, drug accessibility accounts for an irrational choice of medicines and thus exposes oneself to the adverse effects of these medicines.

SM can be unsafe as the misuse of medicines can give rise to side effects and complications [4]. One of the common negative outcomes of the practice of SM is the impact of the irrational use of antibiotics [5]. People are unaware of accurate dosages and complications that profoundly alter what is known as bacterial resistance. A cross-sectional survey conducted in the Sindh province of Pakistan reported that people were unaware of antibiotic resistance associated with inadequate use of antibiotics [6]. Similarly, a report published by the CDC estimated that more than 2.8 million antibiotic-resistance infections occur each year in the United States [7]. Another class of drugs that are commonly abused in self-medicated patients is non-steroidal anti-inflammatory drugs (NSAIDs). This class of drugs is used mainly to alleviate pain, and their misuse may induce severe side effects, including nephrotoxicity and hepatotoxicity [4].

Patients may justify SM for many different reasons that suit their current health needs. A study showed that 37.5% of patients who were admitted to healthcare centers in Edirne, Turkey used medicines without the consultation of a doctor because of a successful experience in the past. Most of them were students with higher education and income. With further investigation, 53% were unaware of antibiotic resistance or toxicity of drugs that occur with overdose. The illiteracy of the public about SM and over-the-counter medicines resulted in the irrational use of drugs [2].

A regional study in the Kingdom of Saudi Arabia conducted in 2017 showed that 49.4% of patients took antibiotics without a prescription because of the long waiting time at the doctor’s office or to schedule an appointment [8]. SM is also significantly linked to the fact that pharmaceutical drugs can be dispensed without a prescription through the malpractice of local pharmacies. People tend to go to these pharmacies for fast check-ups, sometimes without even providing any information about their health issues, and purchase drugs that otherwise must be taken after a consultation with a doctor. Moreover, this study affirmed that about 70% of pharmacies in Makkah were unaware of the fact that dispensing antibiotics without a prescription was illegal [9]. Another study in Jeddah confirmed that 97% of pharmacies dispense many other medicines without a prescription, such as antipsychotics [10]. All in all, these factors highlight serious issues that can lead patients to avoid a doctor’s consultation and thus encourage the behavior of SM.

The previous implications addressed SM among different demographics in the public and should stress this alarming health issue and not regard it merely as the malpractice of different entities. The mentioned regional studies provided sufficient evidence about how SM has been commonly practiced and held in some settings, such as pharmacies. However, we lack evidence of how prevalent SM is in the general public and what factors can be associated with SM. In this study, we aimed to assess the practice and awareness of SM in the general public. Thus, we utilized a questionnaire-based survey to analyze SM awareness and practice among the general population in Jeddah and Makkah. In addition, we examined the impact of demographic variables, such as educational level, economic status, age, etc., on SM practice.

## Materials and methods

Study design, area, and settings

This study was conducted after getting the Institutional Review Board (IRB) approval of ethics at the King Abdullah International Medical Research Center in Jeddah, Saudi Arabia. The IRB number is SP20/040/J. The study was conducted among the general public of Jeddah and Makkah, Saudi Arabia to assess the awareness and practice of SM. A cross-sectional survey (A 1) using a structured and validated questionnaire was chosen for this study. The inclusion criteria of this study were to include Jeddah and Makkah's general populations, all different nationalities, and both genders as well. Participants below the age of 18 years old and with mental and cognitive instability were excluded.

Identification of study participants

*Sampling Technique* 

It was originally designated to use cluster random sampling. Both cities were divided into sub-areas, and a random selection of some of these sub-areas was chosen. Finally, dividing the sample size proportionately among the selected areas followed. However, because of the implications and social constraints of the coronavirus disease 2019 (COVID-19) pandemic, the sampling technique was changed to convenience sampling, where an online questionnaire-based survey was distributed via social media platforms.

Sample Size

The sample size was calculated using CheckMarket software (Checkmarket NV, Turnhout, Belgium). The population of individuals in both Jeddah and Makkah cities was estimated to be about 5,034,981. The required sample size was extrapolated at a 95% confidence level with an estimated 50% response distribution and a margin of error of ±5%. The required minimum sample size was initially set to 385, but to account for the 5% non-response rate, the final sample size was determined to be 404.

Data collection process

The survey was created in a questionnaire format that was also suitable for online access. The questionnaire was validated by experts in medical education and healthcare as well as content validity. In addition, the questionnaire and the consent form were first constructed in English and then translated into Arabic to include individuals whose preferred language is Arabic. The authors of this study distributed the survey through social media platforms with informed consent at the beginning of the survey.

The questionnaire involved two sections; the first section, which consisted of 11 questions, addressed demographic characteristics, such as age, gender, social status, level of education, monthly income, occupation, and health insurance. The second section assessed the awareness and practice of SM, which was subdivided into three components. First, the questions were intended to evaluate the attitude and decisions of the participants about the symptoms of the most recent incidence of illness and its sequence of events. This approach was also utilized to evaluate the most recent behavior of the participants. In contrast, using other approaches such as specifying a limited timeframe would possibly make participants question their memory and consequently reduce the credibility of the responses. In addition, participants were required to list the medicines they consumed as a part of their SM practice, the source of the medicines, and whether there were any side effects indicated to complete the sequence of events. 

Second, participants demonstrated the extent of practicing SM as it might lead to a false sense of expertise in medicine. This might appear as simply advising a relative or friend, where in fact, it is diagnosing, prescribing, or even dispensing a drug without any actual medical knowledge. The final part was implemented to evaluate the participants' attitudes and awareness of whether there are benefits to SM. Finally, while the required sample size was estimated to be 404, 642 responses were collected. This allowed for more assessment of the issue as well as excluding any undesired responses.

Data analysis

Data was downloaded from the Google Form website and stored in Microsoft Excel. It was then transferred and analyzed by Statistical Package for the Social Sciences (SPSS), Version 20.0 (IBM Corp., Armonk, NY).

The numerical variable (age) was presented as median and Interquartile range (IQR). Qualitative variables, like gender, educational level, and marital status, were presented by frequency, and percentage. Chi-square or Fisher exact was used for the comparison of two categorical variables where appropriate. A simple bar graph was used for the graphical representation of categorical variables. A p-value <0.05 was defined as significant.

## Results

Demographic characteristics of study participants

A total of 642 participants completed the online-based survey, but only 472 responses were considered according to the inclusion criteria mentioned in the Methods section. The highest age of the participants was 66 years old with a median age of 32 years old. Of all the participants, 246 (51.2%) were males. About ninety-six percent were from Saudi Arabia while the rest of the participants were from the Philippines (0.6%), Egypt (0.4%), and other nationalities (3.2%). The online-based survey only included participants who are residents of Makkah 105 (22.2%) and Jeddah 367 (77.8%). More, the majority of the included participants had a bachelor’s degree, 300 (63.6%). There were also other different degrees: primary school three (0.6%), middle school six (1.3%), high school 103 (21.8%), higher education (either master’s or Ph.D.) 48 (10.2%), and other levels of educations were only 11 (2.3%) participants. The monthly income was also a key variant in this study. There were three classes of individuals according to their monthly income: less than SAR 5,000 was the income of 220 (46.6%) respondents, between SAR 5,000 and 10,000 was selected as the income of 73 (15.5%) respondents, and more than SAR 10,000 was selected by 179 (37.9%) of the respondents. Other important demographic variants were evaluated (Table 1).

**Table 1:** Demographic characteristics of participants of the general public in Makkah and Jeddah

		Median	IQR
Age (years)		32	22- 45
		Frequency (n=472)	Percentage %
Gender			
	Male	246	52.1
	Female	226	47.9
Ethnic background			
	Saudi Arabia	452	95.8
	Egypt	2	0.4
	Philippines	3	0.6
	Others	15	3.2
The City of Residence			
	Jeddah	367	77.8
	Makkah	105	22.2
Educational level			
	None	1	0.2
	Primary school	3	0.6
	Middle school	6	1.3
	High school	103	21.8
	Bachelor's degree	300	63.6
	Higher education (Masters or PhD)	48	10.2
	Others	11	2.3
Monthly income (SAR)			
	<5,000	220	46.6
	5,000-10,000	73	15.5
	>10,000	179	37.9
Employment			
	Healthcare worker (e.g., doctor, nurse or pharmacist)	47	10.0
	Not a healthcare worker (e.g., teacher, engineer, self-employed, business)	195	41.3
	Unemployed	60	12.7
	Student	140	29.7
	Retired	30	6.4
The presence of a healthcare worker relative			
	Yes	275	58.3
	No	197	41.7
The presence of health insurance			
	Yes	178	37.7
	No	294	62.3
Social status			
	Married	274	58.1
	Single	187	39.6
	Divorced	10	2.1
	Others	1	0.2
Living condition			
	Live with family	431	91.3
	Live alone	29	6.1
	Shared apartment	12	2.5

Symptoms that participants had last time they were feeling unwell

When answering about their latest symptoms when they were sick, most of the participants experienced different types of pain (22.0%), followed by fever (20.8%), and both runny nose and headache equally (19.1%). The remainder of other symptoms were also assessed in the survey, such as cough (13.1%), diarrhea (10.6%), unknown symptoms (9.7%), and breathlessness (6.6%). In addition, there was only a small portion (6.6%) of other symptoms that were not asked about in the survey (Table 2).

**Table 2:** Symptoms that participants had last time they were sick

Symptoms	Frequency (n=472)	Percentage%
Fever	98	20.8
Runny Nose	90	19.1
Pain	104	22.0
Diarrhea	50	10.6
Breathlessness	31	6.6
Headache	90	19.1
Cough	62	13.1
I do not know	46	9.7
Other Symptoms	31	6.6

Means by how participants sought help for their latest symptoms and the reasons for avoiding a visit to a doctor

**Figure 1:**
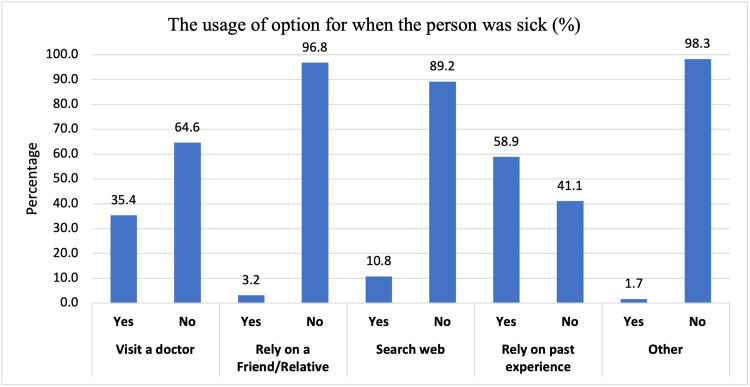
The usage of option when the person was sick (%) Bar graph of the attitude of participants towards their latest symptoms

There was about thirty-five percent of the participants who visited a doctor. However, the data showed that the majority (58.9%) relied on their own experience, only 15 (3.2%) participants relied on advice from a friend or relative, and about 11% searched the web for their symptoms. Furthermore, people who did not visit a doctor had the commonest belief (26.1%) that they did not need a doctor to investigate their symptoms. Other reasons that made people not visit a doctor were due to the long wait (8.5%), avoiding expense (5.1%), and lack of trust in doctors (4.7%) (Table 3) (Figure 1). 

**Table 3:** The attitude of participants towards their latest symptoms and the reasons for avoiding a visit to a doctor

When you were sick, did you	Frequency (n=472)	Percentage %
Visit a doctor	167	35.4
Rely on a Friend/Relative	15	3.2
Search web	51	10.8
Rely on past experience	278	58.9
Other	8	1.7
If you did not visit a doctor, the reason was	n(472)	%
Expense	24	5.1
Long wait	40	8.5
Lack of trust	22	4.7
I have visited a doctor	119	25.2
I don’t need a doctor	123	26.1
Other	17	3.6

Sources of unprescribed medicines among self-medicated participants, the class of these medicines, and the relevant side effects

The highest percentage (37.3%) of the respondents reported that they bought a medicine without a prescription from a pharmacy. Also, to a lesser degree, 30.3% of leftovers were the source of their medicines, and 0.6% of their medicines were provided by a relative or friend. However, 83 (17.6%) of them used medication with a prescription. Moreover, the use of analgesics was the most used drug (65.6%), followed by antipyretics (23.7%). However, antibiotics and herbal medicines had fewer use rates, at 15.3% and 15.9%, respectively. The participants reported that there were side effects that accompanied the medicines they had, in which most of the time (6%), they had dizziness. Participants had other side effects, such as vomiting (2.5%), diarrhea (1.9%), blurred vision (0.8%), and other side effects (2.5%) (Table 4). 

**Table 4:** Source of unprescribed medicines among self-medicated participants, the class of medicines, and relevant side effects

If you used a medicine without a prescription, did you	Frequency (n=472)	Percentage %
Buy it from a pharmacy	176	37.3
Use leftovers	143	30.3
Take it from a relative or friend	3	0.6
I have used a medication with a prescription	83	17.6
I did not use any medication	92	19.5
Other	12	2.5
What was the medication?	n(472)	%
Pain killers	309	65.5
Antibiotic	72	15.3
Antipyretic	112	23.7
Herbs	75	15.9
Other	45	9.5
Side effects of medication (if present):	n(472)	%
Vomiting	12	2.5
Diarrhea	9	1.9
Dizziness	27	5.7
Blurring vision	4	0.8
No side effects	349	73.9
I do not know	84	17.8
Other	12	2.5

The medications that were suggested by the participants to others and their corresponding symptoms

Of all the participants, more than half (270, 57.2%) suggested medication to a relative or a friend. Their relatives or friends’ symptoms included headache (24.4%), pain (21.8%), fever (11.0%), cough (8.1%), breathlessness (1.5%), and other symptoms only (4.0%). The most suggested medication was analgesics (44.7%). Also, participants suggested other medications to others, such as natural herbs (12.7%). Others were antipyretics (11.4%), antibiotics (4.4), and others (3.6%) (Table 5). 

**Table 5:** The medications suggested by the participants to others and the corresponding symptoms

	Frequency (n=472)	Percentage %
Have you ever suggested a certain medication to a relative or a friend?	270	57.2
What were the symptoms?		
Fever	52	11.0
Pain	103	21.8
Breathlessness	7	1.5
No symptoms	230	48.7
Headache	115	24.4
Cough	38	8.1
Other	19	4.0
And what was the medication?		
Pain killers	211	44.7
Antibiotic	21	4.4
Antipyretic	54	11.4
No medication	186	39.4
Natural Herb	60	12.7
Other	17	3.6

The awareness of self-medication among the participants

**Figure 2:**
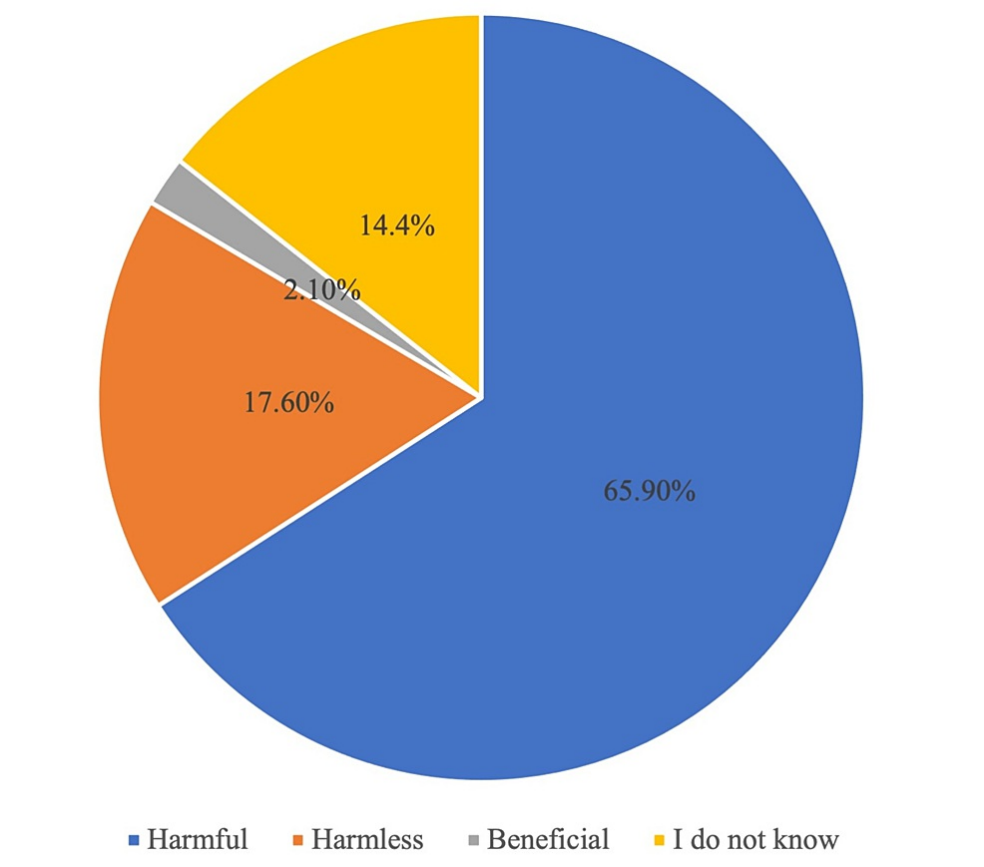
The awareness of self-medication among the participants

The awareness of SM among the general public in Makkah and Jeddah was simply assessed by asking whether they view this practice as harmful, harmless, beneficial, or do not know. Most of them (65.9%) asserted that it was harmful (Figure 2). Also, there was a high percentage (17.6%) of those who regarded SM as a harmless act while only (2.1%) believed it was beneficial and (14.4%) did not have any knowledge about the practice of SM (Table 6). 

**Table 6:** The awareness of self-medication among the participants

	Frequency (n=472)	Percentage %
Harmful	311	65.9
Harmless	83	17.6
Beneficial	10	2.1
I don't know	68	14.4

## Discussion

The practice of self-medication (SM) is prevalent among people worldwide [11]. A myriad of reasons allows people to self-medicate, being unaware of the consequences and undermining them. The reasons range in sources from personal to societal why SM is a common practice as reported by other different studies [12,13]. In light of the literature concerning SM, a cross-sectional survey was utilized to achieve the aim of this study. This study aimed to assess the awareness and practice of SM in the general public of Jeddah and Makkah. The data suggested that there was an incongruity between the public's viewpoint and their practice of SM. Although the majority of the participants (65.9%) believe that SM is harmful, they are inclined to self-medicate on themselves and others when circumstances are not convenient for them to consult a doctor.

As in Thapa et al.’s study, it was expected that the most experienced symptom was pain, and thus pain killers were mostly sought out and recommended by participants [4]. When people experience minor pain, they habitually make an irrational judgment that this does not need a consultation from the doctor. However, the downside resides in the illiteracy of the right doses of the medications taken for that pain. Added to that, sometimes, other symptoms can accompany pain, but the patient is unaware of the full medical issue. Someone with peptic ulcer disease and a stomachache is an example [14]. 

Unlike previous local studies that investigated certain classes of drugs only, this study provided a broader range of medications. In comparison with Alrasheed’s study, antipyretics and antibiotics come in next for the practice of SM [8]. There was not any significant data that suggested major side effects per se. However, although about 74% of the participants reported no side effects from the medications they had without the consult of a healthcare professional, the data made it clear that there was a proportion of exposing oneself to different side effects.

Moreover, there were reasons reported to be the main culprit of avoiding a physician's medical advice. Most of the participants (26.1%) stated that they did not need a doctor. This could be collectively due to the familiarity with the symptoms, which was the highest response (58%), easy access to community pharmacy, and leftovers. This was a similar finding of other studies which concluded that relying on previous experience was the highest source of medical advice [2,15]. Still, other studies emphasized the contribution of other reasons [16-18].

In addition, the high cost of making an appointment was one of the reported reasons. It was anticipated to have to do with the low monthly income of the participants; 46.6% were in the lower quadrant of the monthly income. Regardless, when running a logistic regression on the data, there was an insignificant association between the monthly income and avoiding a visit to the doctor (A 2).

This study contributed to a clearer understanding of SM because it focused more on the general public's perspective rather than the pharmacists’. Our data indicated that pharmacies were the main source of drugs in comparison to Al-Mohamadi et al.'s study [10]. Another important source was a medication recommended to a relative or friend; 57.2% of the respondents. This also indicated the public's indifference to their well-being and the health of others as well. The contradiction between the public's opinion and the actual behavior towards SM implies the importance of exploring the incentives of such behavior.

Lastly, due to the COVID-19 pandemic and its social restrictions, the sampling technique was changed from cluster random sampling to convenient sampling which is less reliable. In addition, the dominant ethnic background of the participants (95.8%) was Saudi Arabia, which could not represent the diversity of the population. Despite these limitations, the results of this study are nonetheless valid for answering our research questions.

## Conclusions

Research reported in this study explored the potential risks associated with SM and analyzed the findings of a cross-sectional study conducted in Jeddah and Makkah to assess SM awareness. Despite 65.9% of the general public believing that SM is harmful, 65.9% practiced it anyway. This contradiction between perception and behavior suggests that people have various reasons for self-medicating, such as convenience, lack of access to healthcare, or financial constraints. Furthermore, the common health conditions for which people preferred SM can vary, such as pain, fever, runny nose, headache, cough, diarrhea, and breathlessness. These are often mild and self-limiting conditions that may not require medical care. However, the misuse of medicines to treat these conditions can lead to antibiotic resistance, adverse drug reactions, and other complications. Thus, it is vital to understand the incentives for SM and promote responsible self-care practices. Through education and awareness programs, the public can make informed health decisions and better understand the risks associated with SM. It is also vital that policymakers address the underlying causes that may motivate people to self-medicate, such as a lack of healthcare infrastructure and affordability issues. Avoiding the adverse effects of SM will ensure less burden on the healthcare system and the individual's overall well-being.

## Figures and Tables

**Figure 3:**
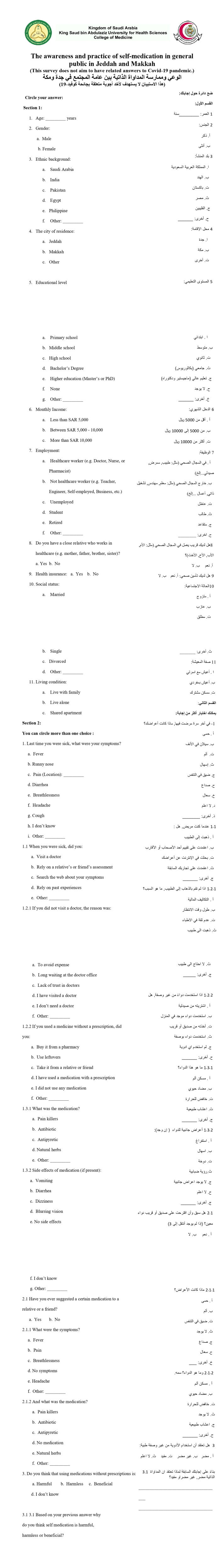
Survey of the study, showing the Arabic and English versions.

## Appendices

**Table 7:** The association between the attitude of participants towards their latest symptoms and monthly income SAR: Saudi Arabian Riyal

	Yes		No		p-value	OR	95% CI	
When you were sick, did you: Visit a doctor								
<5,000 SAR	61	27.7%	159	72.3%		1		
5,000-10,000 SAR	32	43.8%	41	56.2%	0.01*	0.49	0.28	0.85
>10,000 SAR	74	41.3%	105	58.7%	<0.001*	0.54	0.36	0.83
When you were sick, did you: Rely on a Friend/Relative								
<5,000 SAR	11	5.0%	209	95.0%		1		
5,000-10,000 SAR	2	2.7%	71	97.3%	0.53**	1.87	0.40	8.63
>10,000 SAR	2	1.1%	177	98.9%	0.03*	4.66	1.02	21.29
When you were sick, did you: search web								
<5,000 SAR	33	15.0%	187	85.0%		1		
5,000-10,000 SAR	8	11.0%	65	89.0%	0.39*	1.43	0.63	3.26
>10,000 SAR	10	5.6%	169	94.4%	<0.001*	2.98	1.43	6.23
When you were sick, did you: Rely on past experience								
<5,000 SAR	140	63.6%	80	36.4%		1		
5,000-10,000 SAR	38	52.1%	35	47.9%	0.08*	1.61	0.94	2.75
>10,000 SAR	100	55.9%	79	44.1%	0.11*	1.38	0.92	2.07
When you were sick, did you: Other								
<5,000 SAR	4	1.8%	216	98.2%		1		
5,000-10,000 SAR	2	2.7%	71	97.3%	0.63*	0.66	0.12	3.67
>10,000 SAR	2	1.1%	177	98.9%	0.57*	1.64	0.30	9.05
